# Epidemiology and Molecular Virus Characterization of Reemerging Rabies, South Africa

**DOI:** 10.3201/eid1312.070836

**Published:** 2007-12

**Authors:** Cheryl Cohen, Benn Sartorius, Claude Sabeta, Gugulethu Zulu, Janusz Paweska, Mamokete Mogoswane, Chris Sutton, Louis H. Nel, Robert Swanepoel, Patricia A. Leman, Antoinette A. Grobbelaar, Edwin Dyason, Lucille Blumberg

**Affiliations:** * National Institute for Communicable Diseases of the National Health Laboratory Service, Johannesburg, South Africa; † University of the Witwatersrand, Johannesburg, South Africa; ‡ Onderstepoort Veterinary Institute, Pretoria, South Africa; § University of Pretoria, Pretoria, South Africa; ¶ Limpopo Department of Health and Social Development, Polokwane, South Africa; # University of Limpopo, Polokwane, South Africa; ** Limpopo Department of Agriculture, Polokwane, South Africa

**Keywords:** Rabies, South Africa, outbreak, encephalitis, Limpopo, research

## Abstract

The incidence of dog rabies in Limpopo Province, South Africa, increased from 5 cases in 2004 to 100 in 2006. Human rabies had last been confirmed in 1981, but investigations instituted after an index case was recognized in February 2006 identified 21 confirmed, 4 probable, and 5 possible human cases between August 5, 2005, and December 31, 2006. Twelve of these case-patients were identified retrospectively because the diagnosis of rabies was not considered: 6 of these patients consulted a traditional healer, 6 had atypical manifestations with prominent abdominal symptoms, and 6 of 7 patients tested had elevated liver enzyme activity. Molecular genetic analysis indicated that outbreak virus strains were most closely related to recent canine strains from southern Zimbabwe. Delayed recognition of the human cases may have resulted from decreased clinical suspicion after many years of effective control of the disease and the occurrence of atypical clinical presentations.

## Abstract 2

Late identification of an outbreak of human rabies in Limpopo Province led

Despite the availability of effective human and animal vaccines against rabies, and other measures for its control, rabies continues to account for at least 55,000 human deaths each year, mainly in the developing countries of Africa and Asia (*1*,*2*). In these countries, most human rabies infections result from exposure to infected dogs, by bites, scratches, and mucosal exposures (*3*). Rabies vaccination of animals and postexposure prophylaxis (PEP) for humans is prohibitively expensive for most African governments, and it has long been contended that the effects of rabies are underestimated in Africa (*4*).

Typical furious rabies occurs as an encephalitis, often with characteristic features such as hydrophobia and salivation, following a brief, nonspecific, febrile prodrome. Less commonly, rabies may occur in the paralytic form in which characteristic clinical features may be absent (*3*,*5*).

Limpopo is the northernmost province in South Africa and shares borders with Zimbabwe and Botswana. To the East, Limpopo is flanked by the Kruger National Park and Mozambique (Figure 1). The climate is variable with temperate and subtropical areas, and most of the population live in rural villages and subsist by farming maize and livestock.

**Figure 1:**
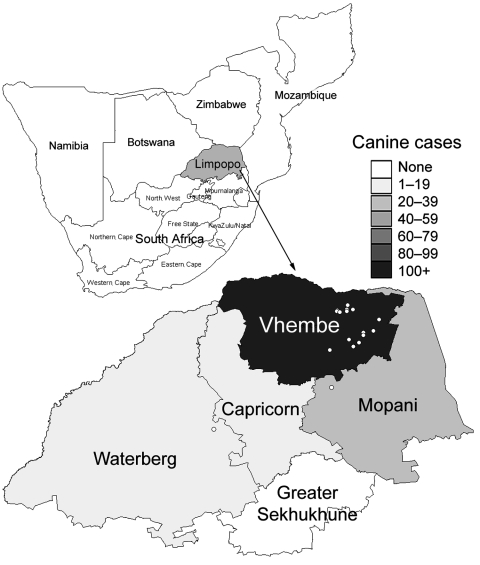
Provinces of South Africa, and neighboring countries. Inset shows a choropleth map of the number of confirmed dog rabies cases by district in Limpopo Province in 2005–2006 and the location of human cases (4 case-patients, for whom coordinates of place of residence were unavailable, were excluded).

Since the 1970s, most human rabies cases in South Africa have occurred in KwaZulu-Natal Province, where the major animal vector is the domestic dog (*6*). Human rabies is much less common in areas such as Limpopo Province, where the major animal vectors are wild animals such as the black-backed jackal species (*Canis mesomelas),* because these animals are less likely to come into contact with humans. Before this report, the most recent 2 laboratory-confirmed human rabies cases in Limpopo Province occurred in 1980 and 1981 (R. Swanepoel, pers. comm.). We describe the epidemiologic, clinical, and viral molecular features of an outbreak of rabies in Limpopo Province, South Africa, in 2005–2006.

## Methods

### Animal Rabies Surveillance

Brain specimens from all animals with suspected rabies in South Africa were submitted to the Rabies Reference Laboratory at Onderstepoort Veterinary Institute, Pretoria. Routine data collected included species, location of case-patient, and date of specimen collection. Archived data on confirmed animal rabies cases from Limpopo since January 1994 were reviewed. An animal case was defined as any case that was laboratory confirmed by fluorescent antibody test (FAT) (*7*) or virus isolation after specimen inoculation into suckling mice and monitoring for signs of rabies infection over 28 days.

Dog vaccine coverage was estimated as the number of doses of vaccine administered to dogs per year divided by the estimated dog population. A ratio of 7 persons to 1 dog was used to estimate the dog population based on unpublished survey data (E. Dyason, pers. comm.). Estimates of the human population by district were supplied by Statistics South Africa (Stats SA, Pretoria, South Africa).

### Human Rabies Surveillance

Human rabies is notifiable in South Africa (Health Act No. 63, 1977); diagnostic specimens from suspected case-patients were submitted to the Special Pathogens Unit at the National Institute for Communicable Diseases, Johannesburg. The diagnosis of rabies was confirmed by using FAT on brain tissue (*7*); by using a heminested reverse transcriptase–PCR (RT-PCR) of saliva (*8*); or by isolating virus from brain, saliva, and cerebrospinal fluid (CSF) specimens as described above. Serum and CSF specimens received were tested for antirabies antibodies, and CSF specimens were tested for viral RNA by RT-PCR. Serologic testing was performed by using indirect immunofluorescence (*9*).

### Epidemiologic Investigation of Human Cases

The study team visited hospitals in the outbreak area in February 2006. Potential cases of rabies (meeting the clinical case definition) in the previous 12 months were identified by clinician interviews, and prospective active surveillance was introduced for new suspected rabies cases.

Data were collected on a standardized data collection form and included demographic data, clinical and laboratory features, history of animal exposure, management of the initial bite exposure, and patient outcomes (Table). Data were obtained by review of clinical records and interview of attending clinicians. For 3 probable cases, no clinical records or laboratory results were available, and data were obtained only by interview of the attending clinician. Data on the cost and numbers of doses of vaccine and immunoglobulin distributed in Limpopo Province were obtained from relevant manufacturers.

**Table:** Clinical and laboratory features of confirmed, probable, and possible human rabies cases, Limpopo Province, South Africa, 2005–2006

Characteristic	No. confirmed cases/total (%)	No. probable cases/total (%)	No. possible cases/total (%)	Total
Clinical features				
Hypersalivation	19/21 (90)	4/4 (100)	1/2 (50)	24/27 (88)
Agitation	14/21 (67)	2/4 (50)	0/2 (0)	16/27 (59)
Weakness or paralysis	14/21 (67)	3/4 (75)	2/2 (100)	19/27 (70)
Fever	14/21 (67)	1/4 (25)	1/2 (50)	16/27 (59)
Hallucinations	11/21 (52)	4/4 (100)	1/2 (50)	16/27 (59)
Confusion	12/21 (57)	0/4 (0)	1/2 (50)	13/27 (48)
Hydrophobia	8/21 (38)	1/4 (25)	0/2 (0)	9/27 (33)
Alternating lucidity and confusion	8/21 (38)	0/4 (0)	1/2 (50)	9/27 (33)
Aggression	6/21 (29)	1/4 (25)	0/2 (0)	7/27 (26)
Vomiting	6/21 (29)	1/4 (25)	1/2 (50)	8/27 (30)
Spasms	5/21 (24)	0/4 (0)	0/2 (0)	5/27 (19)
Convulsions	5/21 (24)	0/4 (0)	1/2 (50)	6/27 (22)
Abdominal distension	4/21 (19)	1/4 (25)	0/2 (0)	5/27 (19)
Pain at the bite site	4/21 (19)	0/4 (0)	1/2 (50)	5/27 (19)
Diarrhea	2/21 (10)	1/4 (25)	0/2 (0)	3/27 (11)
Insomnia	2/21 (10)	1/4 (25)	0/2 (0)	3/27 (11)
Laboratory results				
Elevated leukocyte count (>10 x 10^9^ cells/L)	9/19 (47)	4/4 (100)	0/2 (0)	13/25 (50)
Elevated urea (>7 mmol/L)	11/19 (61)	2/4 (50)	0/2 (0)	13/25 (50)
Elevated creatinine (>100 μmol/L)	1/19 (5)	1/4 (25)	0/2 (0)	2/25 (1)
Elevated total bilirubin (>21 μmol/L)	0/5 (0)	0/4 (0)	0/1 (0)	0/10 (0)
Elevated conjugated bilirubin (>6 μmol/L)	0/5 (0)	0/4 (0)	0/1 (0)	0/10 (0)
Elevated alkaline phosphatase (>120 IU/L)	3/5 (60)	3/4 (75)	1/1 (100)	7/10 (70)
Elevated gamma glucosyl transferase (>35 IU/L)	2/5 (40)	0/4 (0)	0/1 (0)	2/10 (20)
Elevated alanine transaminase (>40 IU/L)	1/5 (20)	2/4 (50)	0/1 (0)	3/10 (30)
Elevated aspartate transaminase (>40 IU/L)	2/5 (40)	2/4 (50)	0/1 (0)	4/10 (40)

#### Case Definitions

*Clinical case*. A clinical case-patient was defined as any person who died after January 1, 2005, and who resided in Limpopo Province before onset of illness with 1 of the following clinical symptoms—delirium, hydrophobia, salivation, acute psychosis, acute flaccid paralysis, muscle spasms, convulsion or respiratory paralysis—and with no other identified cause of death.

*Possible case*. A possible case-patient was defined as a person who met the clinical case definition, but whose case was not laboratory confirmed, and who had no documented history of animal exposure.

*Probable case*. A probable case-patient was defined as a person who met the clinical case definition, but whose case was not laboratory confirmed, and who had history of exposure to a suspected rabid animal.

*Confirmed case*. A confirmed case-patient was defined as a person who met the clinical case definition and had laboratory-confirmed rabies.

### Molecular Analysis of Viruses Obtained from Animal and Human Rabies Case-Patients

After viral RNA underwent extraction and RT-PCR (*10*,*11*), the amplicons obtained were purified with a commercial kit (Wizard SV Gel and PCR Clean-Up System, Promega, Madison, WI, USA) and sequenced bidirectionally on an ABI377 automated DNA sequencer (Applied Biosystems, Foster City, CA, USA) with the G/L primer set. A 592-bp nucleotide portion of the cytoplasmic domain of the glycoprotein and the G-L intergenic region of the virus isolates included in the study sample were aligned in ClustalW (*12*). A phylogenetic tree was constructed with the neighbor-joining method (*13*) in MEGA (Molecular Evolutionary Genetics Analysis) software version 2.1 (*14*), and 1,000 replications. The phylogenetic tree was visualized with TreeView (*15*).

All available isolates from humans and a panel of dog rabies virus isolates from Vhembe were selected (Appendix Table). Virus isolates from other provinces in South Africa and neighboring countries were also included in phylogenetic reconstruction of the molecular epidemiology.

## Results

### Animal Rabies Cases

**Figure 2:**
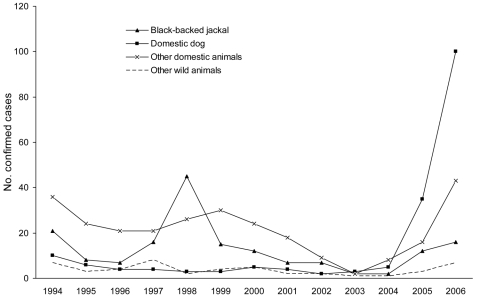
Laboratory-confirmed animal rabies cases, Limpopo Province, South Africa, 1994–2006.

From 1994 through 2004, 8 to 76 laboratory-confirmed animal rabies cases were identified from Limpopo Province annually. Most of these cases were in *C. mesomelas* (black-backed jackal) and in livestock (mainly cattle) (Figure 2). *C. mesomelas* case numbers increased to 12 in 2005 and 16 in 2006.

**Figure 3:**
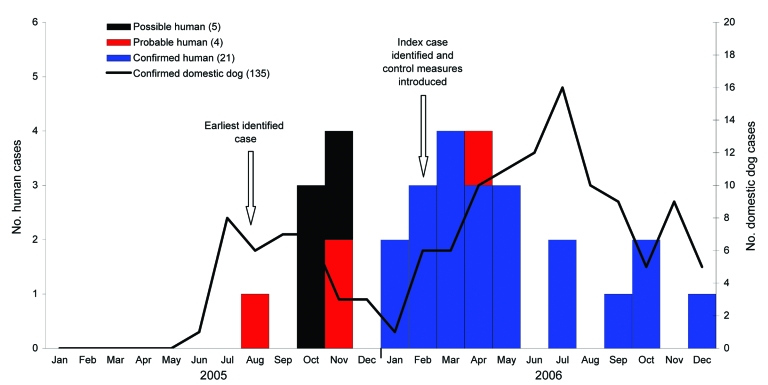
Numbers of possible, probable, and confirmed human cases and laboratory-confirmed domestic dog rabies cases by month of diagnosis, Limpopo Province, South Africa, 2005–2006.

Fewer than 10 rabies cases per year were reported from Limpopo in domestic dogs (*Canis familiaris*) from 1994 through 2004 (Figure 2). The number of laboratory-confirmed dog rabies cases increased markedly from 5 in 2004 to 35 in 2005 and 100 in 2006 (Figure 3). Most dog cases (106/135, 79%) in 2005 and 2006 came from the Vhembe District. The mean estimated dog vaccination coverage in Vhembe District from 1997 through 2005 was 39%; annual coverage estimates fluctuated but ranged from 4% to 60% (E. Dyason, pers. comm.).

### Human Rabies Cases

#### Detection of the Outbreak

Rabies was confirmed by RT-PCR testing on a saliva specimen from the index case-patient, a 10-year-old boy who was brought to the hospital on February 7, 2006 (Figure 3). Twelve patients with encephalitis that met the case definition were identified retrospectively, and rabies was confirmed by IFA for 2 of these patients for which brain tissue was available and by RT-PCR of saliva for a further 2 patients. The earliest identified case-patient was a 9-year-old boy who was admitted in August 2005 with a history of dog bite. An admission CSF specimen, submitted for rabies diagnosis, was found to be negative by both RT-PCR and antibody testing.

#### Description of the Outbreak

From January 1, 2005, through December 31, 2006, 21 confirmed, 4 probable, and 5 possible human rabies cases were identified (Figure 3). The earliest identified case-patient became ill on August 5, 2005. The numbers of confirmed cases peaked in March 2006. Case numbers decreased from May 2006, but 1 to 2 cases per month continued to be reported until December 31. Of the 30 case-patients, 28 were from the Vhembe District (Figure 1).

Twenty-seven cases were in children 3–12 years of age (median 9 years). All case-patients were hospitalized. The median duration from admission to death was 4 days (range 1–25 days). All 4 patients who survived >10 days were admitted to intensive care units.

#### Clinical and Laboratory Features of Human Cases

The median incubation period was 8 weeks (range 3–28 weeks) for the 22 case-patients for whom the date of exposure was known. The most common clinical feature observed in patients with confirmed cases was salivation (19/21, 90%), followed by agitation (14/21, 67%), weakness (14/21, 67%), fever (14/21, 67%), and hallucinations (11/21, 52%) (Table).

The median period between when a person first experienced illness and when the person sought healthcare was 2 days (range 0–8 days) in the 19 patients for whom date of onset of symptoms was available. Lumbar puncture was performed on 14 patients. CSF findings were within normal limits for all 11 patients who did not have blood in the CSF specimen. Nine of 19 patients tested (47%) had an elevated leukocyte count (>10 ×10^9^/L), and 11 (61%) of 18 had elevated urea levels (>7 mmol/L). All 4 patients tested for HIV were HIV seronegative. No abnormalities were detected in hemoglobin level, platelet count, or erythrocyte sedimentation rate in any of the 19 patients.

The cluster of 11 case-patients who sought treatment before the index case-patient was identified (excluding the primary case-patient in whom rabies was suspected) was reviewed separately to identify possible reasons for the delayed diagnosis of rabies. Of these patients, 6 reported having consulted a traditional healer before visiting the clinic. Clinical and laboratory data were available for 8 patients: 6 exhibited prominent abdominal symptoms (including abdominal distension in 4, vomiting in 3, and diarrhea in 2), 3 of whom reported consulting a traditional healer. Liver function tests were performed for 7 case-patients; of these, 6 had elevated alkaline phosphatase enzyme levels (>120 IU/L). Clinicians’ differential diagnoses of these cases included viral encephalitis, typhoid, pyrexia of unknown origin, epilepsy, panic attacks, poisoning or toxin exposure, and Guillain-Barré syndrome. Five patients were not asked about possible animal exposures.

For 7 case-patients reported in this outbreak, specimens were not submitted for rabies diagnosis. For all 21 confirmed case-patients, saliva specimens were positive by RT-PCR; for 7 of these case-patients, brain tissue specimens sampled on postmortem examination were also positive by IFA, and for 3 unvaccinated patients, serum specimens were positive for rabies antibodies. Virus isolation was attempted on 7 saliva specimens and was successful for 5. No virus isolation was attempted on brain specimens because all were received in formalin, despite guidelines for submission of samples in glycerol saline. All 7 CSF specimens were collected during the first week of illness and were negative for antirabies antibodies and by RT-PCR. A saliva specimen from 1 patient with typical rabies symptoms, who had been bitten by a dog with suspected rabies, tested negative by RT-PCR. He was classified as a probable case-patient; postmortem brain tissue could not be obtained.

### Management of Exposures

All 24 case-patients who were asked about a history of animal exposure reported an exposure to a potentially rabid dog. All documented exposures were category 3 (high-risk) exposures, i.e., a bite or scratch that drew blood or a lick to mucous membranes or broken skin. Most patients (20/24, 83%) reported a bite, but 3 patients reported scratches only, and 1 reported that the dog had licked and nibbled at mucous membranes. For 22 case-patients with a known date of exposure, 15 (68%) exposures had occurred before the outbreak was identified and control measures were implemented.

Of 16 case-patients for whom site of exposure was reported, half of the exposures (8) were on the lower limb, but exposures were also reported to the upper limb (3), trunk (2), and head and neck (3). Most exposures were to unknown dogs, but 5 of 20 case-patients reported exposure to their own dog. Of the 18 case-patients able to give a history of the management of the original bite exposure, 12 (67%) did not report to a clinic at the time of exposure. All 6 case-patients who sought treatment at clinics received wound cleaning, but only 2 were vaccinated (1 received only 1 dose).

One case-patient, a 4-year-old boy who had been bitten on the left cheek by a dog on September 6, 2006, received antirabies immunoglobulin (Rabigam, National Bioproducts Institute, Pinetown, South Africa) in addition to vaccination with Verorab (Sanofi Pasteur, Lyon, France) within 12 hours of exposure. Details of wound cleaning are unclear, although the wound was not sutured. The patient received antirabies immunoglobulin at the recommended dose of 20 IU/kg, half injected into the wound site and half injected into the deltoid muscle, and rabies vaccine administered into the deltoid muscle on days 0, 3, 7, and 14. Whether this was the deltoid opposite to that used for the immunoglobulin was not known. Rabies developed in the patient on September 23, 2006 (17 days after exposure) and was confirmed by RT-PCR of brain tissue; the patient died on September 25, 2006. Vaccine and immunoglobulin batches were found to meet required potency standards (Z. Goondiwala, Sanofi Pasteur, pers. comm.; C. Rochat, National Bioproducts Institute, pers. comm.).

### Molecular Epidemiology

**Figure 4:**
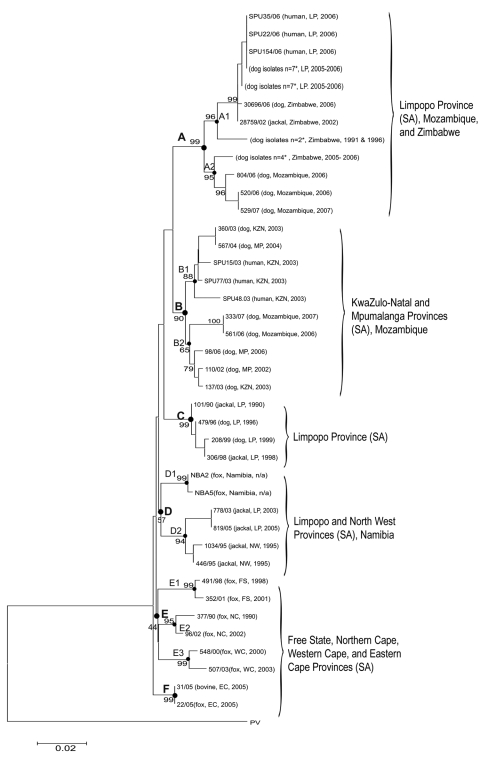
Neighbor-joining tree of canid rabies viruses from humans and animals from Limpopo (LP), Mpumalanga (MP), North West (NW), Free State (FS), Eastern Cape (EC), Northern Cape (NC), KwaZulu-Natal (KZN), and Western Cape (WC) Provinces of South Africa (SA) and neighboring countries of Zimbabwe, Mozambique, and Namibia. The Pasteur virus strain (PV) was used as the reference strain in the sequence alignment. Horizontal scales represent the evolutionary distance; vertical lines are for clarification purposes only. The scale bar indicates nucleotide substitutions per site. Viruses are identified by a laboratory reference number, source animal, locality of origin, and year of isolation. A–F represent virus lineages supported by bootstrap values of >70%; sublineages are indicated numerically. *Identical strains.

Phylogenetic analysis of nucleotide sequences indicated that the viruses originating from humans in the Vhembe area of Limpopo were genetically indistinguishable from those obtained from domestic dogs in the same geographic area (Figure 4). Notably, this cluster represented a new phylogenetic group not previously encountered in Limpopo Province (*16*) and clearly distinct from the viruses isolated from *C. mesomelas* from Limpopo. Outbreak viruses were most closely related to viruses obtained from dogs and jackals across the border in southern Zimbabwe (sublineage A1). A second closely related sublineage (A2) was composed of viruses from southeastern Zimbabwe and western Mozambique, which suggests that a dog rabies cycle exists within South Africa, Zimbabwe, and Mozambique. The inclusion and analysis of rabies virus isolates from other provinces of South Africa and neighboring countries did not suggest any close link with the outbreak viruses.

### Control Measures

Central-point dog vaccination campaigns in villages in the affected area were intensified after identification of the increased numbers of rabies cases in domestic dogs. A community awareness program related to the hazards of dog bites and the importance of timely visits to the clinic for rabies postexposure prophylaxis was established in February 2006. Furthermore, healthcare workers were educated regarding appropriate management of dog bites. Vaccine and immunoglobulin availability was improved by increasing the number of facilities providing the vaccine and by ensuring that patients did not have to pay for treatment. Registers of dog bite cases were implemented in clinics that did not have existing registers in March 2006. All registering staff emphasized the importance of documentation and follow-up for those not returning for all scheduled doses of rabies vaccine.

The combined number of doses of human rabies vaccine (human diploid cell [Mérieux Inactivated Rabies Vaccine, Sanofi Pasteur, Lyon, France], purified Vero cell vaccine [Verorab, Aventis Pasteur], and inactivated chick embryo vaccine [Rabipor, Biovac, Johannesburg, South Africa]) used in Limpopo Province in the public sector increased from 3,000 in 2004 to 6,000 in 2005 and 56,000 in 2006 (R. Watson, Biovac, pers. comm.). Use of antirabies immunoglobulin (Rabigam, National Bioproducts Institute, Pinetown, South Africa) also increased over the same period with ≈100 doses given in 2004, increasing to 500 in 2005 and 2,500 in 2006 (C. Rochat, National Bioproducts Institute, pers. comm.). At a cost of 130 South African rand (R130; US $18) per vaccine dose and R300 (US $43) per immunoglobulin dose, total cost for biologics alone is estimated at 8 million R (≈US $1.1 million) for the year 2006. This figure would be substantially higher with the inclusion of patient costs and other indirect costs.

## Discussion

We describe an outbreak of human rabies in a province of South Africa where rabies had been well controlled for >10 years. Late recognition of this outbreak resulted in delayed implementation of control measures. Although the clinical features of classic rabies have been described as unmistakable (*5*), the diagnosis may be missed due to low index of suspicion and variable clinical features (*17*), as occurred in this outbreak. Cases of rabies may be incorrectly attributed to other causes of pyrexia and confusion common to rural Africa, including cerebral malaria, bacterial infections, and infection with HIV (*18*,*19*).

In this outbreak, the clinical signs and symptoms of the initial case-patients may have been altered due to use of traditional medicines. Of 12 case-patients in whom the diagnosis of rabies was missed, 6 reported having visited a traditional healer before seeking treatment at a hospital. The use of traditional medicines is common in rural settings in South Africa (*20*,*21*) and may result in toxicities, including abdominal and psychiatric symptoms and abnormal liver function test results (*22*). These medicines could have contributed to the atypical manifestations in some cases. In addition, clinicians may have attributed some of the neurologic symptoms to herbal intoxication.

Nevertheless, rabies was in fact suspected in the primary case-patient, identified in August 2005. The diagnosis was not, however, confirmed because an inappropriate specimen (a CSF specimen taken on admission) was submitted. Anti-rabies antibodies in the CSF are not usually detected <1 week after the onset of clinical illness, and RT-PCR results for rabies RNA on CSF may be negative in rabies cases; thus, a negative CSF result does not exclude the diagnosis of rabies (*17*,*18*,*23*). It is therefore recommended that repeated saliva and serum specimens be submitted in addition to CSF and that a postmortem brain specimen be actively sought in all suspected rabies cases (*18*).

Four case-patients who sought treatment at a clinic before identification of the outbreak were not offered PEP, probably because the risk for rabies infection was not considered. Our case series includes 1 child in whom rabies developed despite the administration of seemingly adequate PEP. Possible contributing factors to the development of rabies in this case include the facial location of the wound, possible inadequate wound cleansing, and the fact that all of the immunoglobulin could not be infiltrated into the wound site. The full dose of immunoglobulin should be administered on the first day of PEP and should be infiltrated into the wound (*24*,*25*).

Rabies of the canid biotype has been endemic in *C*. *mesomelas* in Limpopo Province since the 1950s, with occasional spillover to cattle and domestic dogs. Since 1952, several attempts at control have been made, including destroying ≈22,000 dogs in that year, poisoning an estimated 3,900 jackals from 1951 through 1956, and vaccinating 181,414 dogs from 1952 through 1962 (*6*). Despite these efforts, a low incidence of dog rabies was observed in the province in the 1960s. Rabies became a serious problem again in cattle and jackals in the mid 1970s, likely following its reintroduction from Zimbabwe in 1974, and it has remained endemic in jackals with sporadic cases occurring in domestic dogs (*6*).

As in a classic situation, this outbreak in humans followed an outbreak in domestic dogs of the region. Increasing numbers of human rabies cases in Africa have been attributed to increasing numbers in animals, to the mobility of human and animal populations, and to deteriorating infrastructure and resources for rabies control (*4*,*26*). Reasons for the reemergence of canine rabies in Limpopo after many years of effective disease control are unclear. In Zimbabwe, dog rabies cases increased after 1990, after declining vaccination coverage associated with decreased resources and diversion of resources (*27*). Low vaccination coverage in domestic dogs in Limpopo over several years may have led to an accumulation of susceptible animals, which led to the reestablishment of transmission.

The reintroduction of canine rabies into northern KwaZulu-Natal Province in 1976 followed an influx of refugees from Mozambique (*6*). The possible contribution of increased immigration into Limpopo Province from Zimbabwe in recent years is difficult to quantify (*28*). Molecular genetic analysis indicates that the virus isolates from both humans and dogs in this outbreak were most closely related to those from southern Zimbabwe. This finding suggests that the outbreak may have extended across the border from Zimbabwe.

The number of human rabies cases in Limpopo Province decreased after May 2006; no further human cases had occurred as of June 30, 2007. This decrease is likely due to the introduction of coordinated control measures (including aggressive PEP). Although highly effective if administered correctly, PEP is much more costly than vaccination of domestic dogs (*29*,*30*). Unfortunately, dog vaccination is difficult in many developing countries because of high dog turnover rates, shortages of funding and personnel, and competing priorities (*26*,*31*).

The number of reported human rabies cases, particularly in Africa, greatly underestimates the true effects of the disease. Contributing factors include failure to seek treatment at healthcare facilities, failure to make a laboratory diagnosis, and failure to report the disease (*2*,*32*). Our attempts to conduct active case finding through clinician interviews at hospitals in Vhembe District encountered several problems. First, we were unable to review all hospital admissions records because of incomplete record keeping. We also recognize that at least some infected persons may not have visited hospitals and died at home. In addition, epidemiologic data were not available for all cases since several cases were identified retrospectively. An increased awareness of rabies after interventions for control may have contributed to increased case reporting after February 2006; this situation may have affected apparent trends in human case numbers and contributed to the delay in observed decline in dog cases.

This outbreak highlights the fact that rabies is a transboundary disease and can reemerge in areas where successful control programs have been active for many years. Clinicians should consider rabies in the differential diagnosis, especially in cases of fatal encephalitis and submit appropriate specimens for rabies diagnosis. Sustained awareness, together with political and economic commitment to animal and human rabies control programs, particularly the vaccination of dogs, is essential.
